# Human and environmental controls over aboveground carbon storage in Madagascar

**DOI:** 10.1186/1750-0680-7-2

**Published:** 2012-01-30

**Authors:** Gregory P Asner, John K Clark, Joseph Mascaro, Romuald Vaudry, K Dana Chadwick, Ghislain Vieilledent, Maminiaina Rasamoelina, Aravindh Balaji, Ty Kennedy-Bowdoin, Léna Maatoug, Matthew S Colgan, David E Knapp

**Affiliations:** 1 Department of Global Ecology, Carnegie Institution for Science, 260 Panama Street, Stanford, CA USA; 2 GoodPlanet Foundation, Carrefour de Longchamp, 75116 Paris, France; 3 CIRAD, UR105 Forest Ecosystem Goods and Services, TA C-105/D, Campus de Baillarguet, 34398 Montpellier Cedex 5, France & DRP Forêt et Biodiversité, BP 853, Antananarivo, Madagascar; 4 World Wide Fund for Nature, BP 738, Antananarivo, Madagascar

**Keywords:** aboveground carbon density, biomass, carbon stocks, Carnegie Airborne Observatory, CLASlite, LiDAR, REDD, tropical forest

## Abstract

**Background:**

Accurate, high-resolution mapping of aboveground carbon density (ACD, Mg C ha^-1^) could provide insight into human and environmental controls over ecosystem state and functioning, and could support conservation and climate policy development. However, mapping ACD has proven challenging, particularly in spatially complex regions harboring a mosaic of land use activities, or in remote montane areas that are difficult to access and poorly understood ecologically. Using a combination of field measurements, airborne Light Detection and Ranging (LiDAR) and satellite data, we present the first large-scale, high-resolution estimates of aboveground carbon stocks in Madagascar.

**Results:**

We found that elevation and the fraction of photosynthetic vegetation (PV) cover, analyzed throughout forests of widely varying structure and condition, account for 27-67% of the spatial variation in ACD. This finding facilitated spatial extrapolation of LiDAR-based carbon estimates to a total of 2,372,680 ha using satellite data. Remote, humid sub-montane forests harbored the highest carbon densities, while ACD was suppressed in dry spiny forests and in montane humid ecosystems, as well as in most lowland areas with heightened human activity. Independent of human activity, aboveground carbon stocks were subject to strong physiographic controls expressed through variation in tropical forest canopy structure measured using airborne LiDAR.

**Conclusions:**

High-resolution mapping of carbon stocks is possible in remote regions, with or without human activity, and thus carbon monitoring can be brought to highly endangered Malagasy forests as a climate-change mitigation and biological conservation strategy.

## Background

The spatial distribution of carbon stored in the aboveground tissues of vegetation - also known as aboveground carbon density (ACD; units of Mg C ha^-1^) - is a time-integrated expression of ecological and land-use processes ranging from photosynthesis and nutrient cycling to disturbance and climate change. Spatial variation in ACD is also the largest source of uncertainty in monitoring carbon emissions for voluntary carbon offset markets and for developing international action for Reduced Emissions from Deforestation and Forest Degradation (REDD) at national and sub-national levels [1-4]. Mapping the geographic patterns of carbon storage is thus a high priority in scientific, conservation, and resource-management communities.

Carbon mapping efforts have proven challenging for a variety of reasons. Field inventory plots are critically important at local scales, but they are time consuming, costly, and limited by accessibility. As a result, they usually do not capture the variation in ACD that exists throughout the environment [5]. This is particularly true in human-dominated landscapes, or in remote areas that may harbor remaining forests, and these two scenarios have become ubiquitous in many tropical regions [6]. Madagascar is an important example of these challenges; high rates of deforestation and degradation have transformed millions of hectares of dry, mesic and humid tropical forest, leaving highly fragmented landscapes in most regions [7,8]. Remaining forests cover up to 15% of Madagascar, and thus the vast majority of landscapes are partially-vegetated, human-dominated systems [9]. The spatial heterogeneity of vegetation cover and structure in these landscapes has resulted in a poor overall knowledge of their role in storing carbon.

The remaining forests of Madagascar are also often remote, whether by distance or by difficult terrain, and they are among the last strongholds of the unique forest flora and fauna remaining on the island. These remote forests appear partially to fully intact in satellite imagery [9,10], but satellite sensors do not resolve their carbon stocks at a spatial scale sufficient to understand ecological controls over those stocks. As a result, we know little about controls over carbon storage in Malagasy forests - information that is critically needed to support their inclusion in carbon retention strategies to conserve them.

Mapping ACD beyond the reach of field plots is an important step toward resolving regional carbon dynamics in Madagascar and elsewhere. Airborne Light Detection and Ranging (LiDAR), when combined with field plots, has provided spatially contiguous, high-resolution ACD estimates of temperate and tropical forests [e.g., [11-13]]. Airborne systems can readily acquire thousands of hectares of data per day, changing our understanding of environmental controls over carbon stocks. Airborne LiDAR-based mapping of tropical forest ACD has also proven highly precise and accurate, with errors recently becoming indistinguishable from those derived from field measurements [14].

Despite the carbon mapping accuracy, resolution, and extensive coverage provided by airborne LiDAR, these approaches also reach geographic limitations due to cost, so additional methods are required to extend LiDAR-based carbon estimates to even larger regions. Importantly, the pronounced heterogeneity of carbon stocks in Madagascar requires high spatial resolution when extending to the regional or whole-country scale. One approach to extrapolating outside the LiDAR coverage is to stratify the region into narrow vegetation classes, and then to apply additional analyses to account for vegetation losses and gains from deforestation, degradation and land abandonment [15,16]. This has proven useful in regions already containing detailed vegetation maps derived from satellite or other sources, resulting in regionally-integrated uncertainties in total carbon storage of just a few percent or less [17]. However, such highly stratified maps of vegetation type do not exist for many areas of the tropics [2,18], and other means are necessary to extend sampling-based estimates of ACD - whether taken from field, aircraft or satellite measurements - to broader geographic scales. Again, Madagascar serves as an example of the challenges faced when mapping carbon stocks in most tropical regions, where vegetation maps may not exist or may only be based on coarse representations of geology [e.g., [19]]. A more detailed, top-down approach utilizing satellite imagery is required; however, the most widely available optical satellite images from NASA Landsat or MODIS (Moderate Resolution Imaging Spectrometer) do not directly measure carbon stocks. Rather, optical sensors are most sensitive to vegetation type and cover [20], and so spatial extrapolation of LiDAR-based biomass estimates using optical satellite data alone is subject to substantial uncertainty. Synthetic aperture radar (SAR), which is sensitive to aspects of canopy texture and structure, can be related to biomass stocks [21,22], but direct SAR-based metrics of forest biomass are limited by saturation of the signal in vegetation harboring more than 50-70 Mg C ha^-1^, depending upon radar frequency [23,24]. Another option is to evaluate how carbon stocks vary with topography and other geologic drivers mapped with SAR, but relationships between topography or geology and ACD have rarely been explored [25,26].

Here, we present the first large-scale, high-resolution mapping estimates of aboveground carbon stocks in one northern and one southern region of Madagascar. Using airborne LiDAR-based measurements of ACD, calibrated to a network of field plots, we quantitatively assessed which topographic and land-cover factors best predicted ACD across environmental and land-use gradients in each region. We then used this information, combined with spaceborne optical and SAR data, to extend field and LiDAR carbon estimates to the regional level. Our underlying goal was to understand both human and environmental controls over the carbon landscape in some of the most remote portions of Madagascar.

## Results and discussion

**Figure 1:**
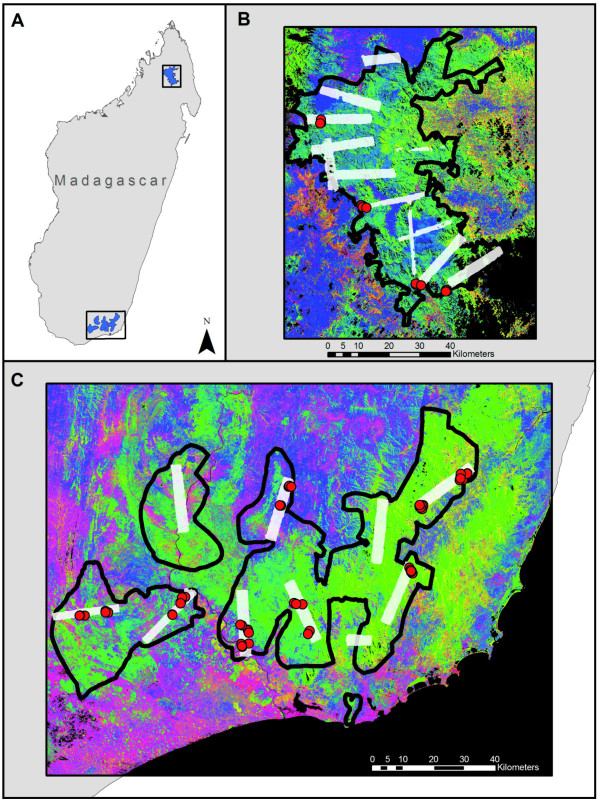
**(A) Madagascar with the 659,592 ha northern and 1,713,088 ha southern study regions**. (B-C) Airborne LiDAR sampling coverage is shown in white boxes. In panels B-C, the background imagery shows the fractional cover of photosynthetic vegetation (PV; green), non-photosynthetic vegetation (NPV; blue) and bare soil (pink-red) derived from CLASlite analyses of Landsat satellite imagery. Field calibration plots are shown as red dots (not to scale).

The northern (N) region encompassed 289,766 ha of core study area within 659,592 ha of total area (Figure 1). The core study area was defined by an ongoing REDD+ project, whereas the total area was defined by our satellite coverage. The N region contained forests broadly classified as mid-elevation (600-2900 m a.s.l.) evergreen humid tropical forest, underlain by basement rock formations [19]. Above 2100 m elevation, forest vegetation transitions to montane Philippia shrubland [19]. Below 900 m, much of the region is deforested, persisting as a spatially complex mosaic of grassland, bare substrate (rock and soil) and patches of woody vegetation protected by local-scale terrain variation (e.g., gullies, steep slopes, etc.). The southern (S) region spanned 495,767 ha of core study area within 1,713,088 ha of total area. This area crosses a strong orographic climate boundary incorporating low- to mid-elevation evergreen humid tropical forests on windward slopes in the east, to deciduous, seasonally dry, or "spiny" forests and shrublands to the west (Figure 1). Geologic substrates vary from basement rock formations in the humid sub-montane forests to basalt and sandstone formations underlying the drier spiny forests. Elevation in the S region reaches a maximum of 1968 m a.s.l., and areas below about 500 m have undergone substantial clearing in the past.

### Field Calibration of Airborne LiDAR

**Figure 2:**
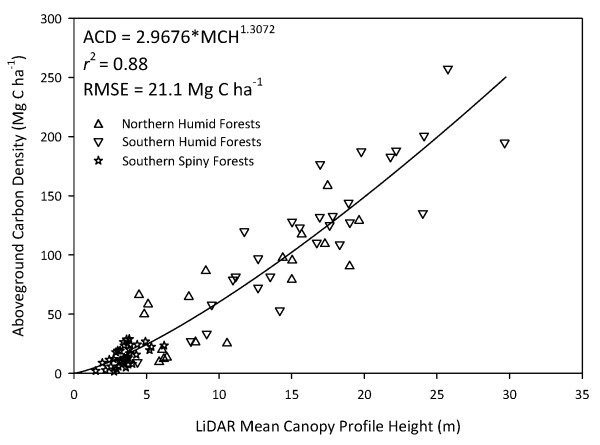
**Relationship between mean canopy profile height (MCH) derived from airborne LiDAR and plot-based aboveground carbon density (ACD) across a wide range of vegetation types and conditions, including degraded forests, in Madagascar**.

Histograms of the plot-based estimates of ACD are shown in Figure S1 (Additional file 1). The mean (± s.d.) ACD of dry forests was 14.5 (8.1) Mg C ha^-1^, with a range of 1.4 to 28.9 Mg C ha^-1^. The mean (± s.d.) ACD humid forest plots was 99.5 (58.5) Mg C ha^-1^, with a range of 9.3 to 257.4 Mg C ha^-1^. Following the processing of the LiDAR data to MCH, we predicted ACD within and across all study regions with high precision (r^2 ^= 0.88) and accuracy (RMSE = 21.1 Mg C ha^-1^) (Figure 2). The power-function scaling coefficient of 1.3 is slightly lower than those derived for forests in the Neotropics, resulting in a nearly linear MCH-to-ACD relationship [discussed in [27]]. An average pixel-level error of 21 Mg C ha^-1 ^compares favorably with past studies showing uncertainties of 40 Mg C ha^-1 ^or more in temperate and tropical forests [11,12,17,28,29].

### Landscape Controls over LiDAR-derived ACD

**Figure 3:**
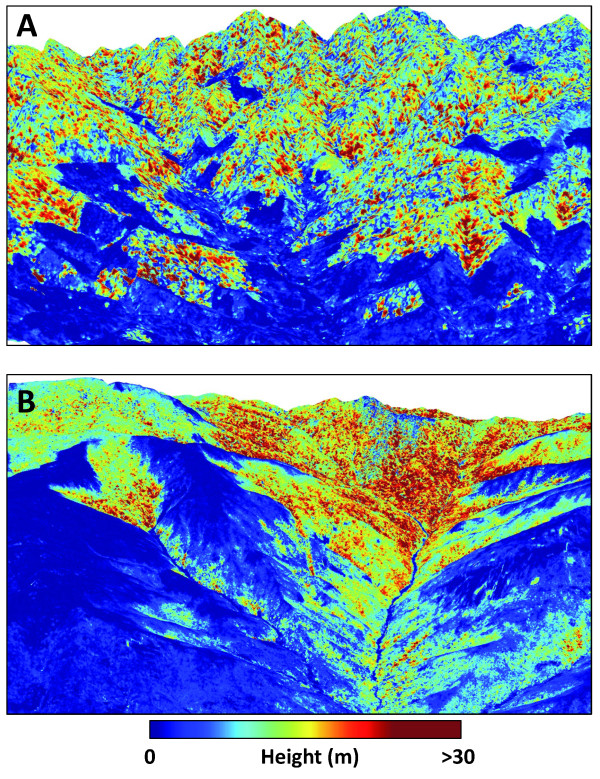
**Example LiDAR-based maps of aboveground carbon stocks highlighting: (A) effects of deforestation on humid, low-elevation forests in the northern region, and (B) a natural gradient in elevation and plant available water, from dry forests with low canopy height in the lowlands to humid forests in the uplands**.

Our airborne LiDAR mapping results revealed the influence of human activity, as well as underlying climatic and physiographic controls, in shaping ACD patterns throughout northern and southern Madagascar. High levels of deforestation have reduced standing carbon stocks in many lowland areas, as evidenced by the sharp decline in LiDAR-derived canopy height in zones of human activity (Figure 3). Yet this signal of human-mediated ACD suppression did not obscure natural ACD gradients controlled by climate and elevation. Forests found at higher elevations were less likely to be deforested or degraded, and were also more likely to contain the tallest forest canopies harboring higher carbon stocks.

**Figure 4:**
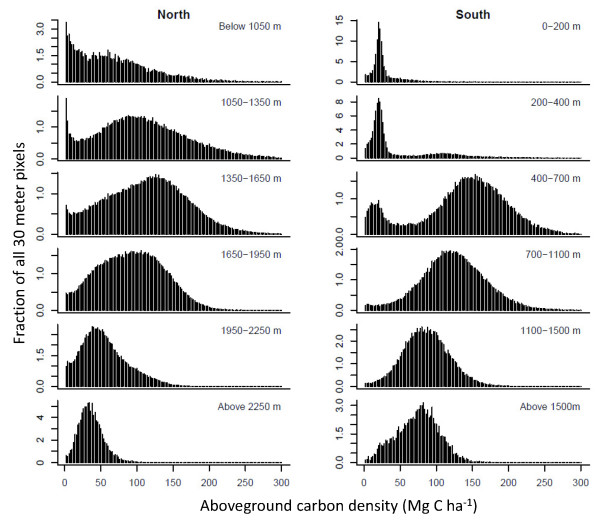
**LiDAR-derived frequency distributions of forest ACD at 30-m resolution by elevation class in the northern and southern regions of Madagascar**. Bimodal distributions in the southern region reflect the contrast between spiny forest (nearly all of which are found < 500 m elevation) and humid forests (which are nearly absent at elevations < 500 m due to heavy deforestation and degradation). At high resolution, ACD can peak at very high values (> 300 Mg C ha^-1^), but we truncate here to facilitate visualization because these high-ACD outliers are much less than 0.1% of any class.

To further quantify the controls over ACD, we spatially integrated the LiDAR-based carbon mapping results in both N and S regions, and found that ACD peaked at intermediate elevations (Figure 4). Moreover, we found major differences in ACD distributions and medians along elevation gradients, and these patterns differed in the N and S regions. In the north, the lowlands (< 1050 m) harbored a highly skewed distribution of carbon storage, resulting from human-driven forest losses leading to low carbon levels; yet with a sufficiently large, high-resolution regional sample size, we also found that ACD can reach very high values in surviving lowland forests. In effect, the right side of the distribution indicates maximum potential carbon stocks in the lowlands, which exceeded 200 Mg C ha^-1^. In contrast to N lowland carbon values, the S region harbored very suppressed ACD levels (Figure 4), and this was due to the drier conditions that support lower-biomass spiny forests as well as extremely high forest loss rates.

In both the N and S regions, aboveground carbon stocks reached their highest median levels at mid-elevation (Figure 4). In the north, we found that ACD peaked in the 1350-1650 m zone, whereas highest median values were closer to 700 m in the south (Appendix 3, Additional file 1). Above these zones of highest carbon storage, both ACD median and variance decreased steadily to much lower and narrower ranges in montane conditions. The sharpest high-elevation decline in ACD occurred above 1950 m in the N region, where forests give way to short-statured Philippia shrublands [19]. In the S region, the decrease in ACD at higher elevations was less pronounced. We note, however, that ACD levels in the S sub-montane (e.g., 1100-1500 m) and montane (> 1500 m) zones were lower and less variable than in the N forests at comparable elevations. Importantly, most ACD distributions were non-normal, suggesting that without very large sample sizes, or carefully distributed sampling, field plots alone may not accurately resolve carbon stock variation.

Deforestation, disturbance and regrowth all imparted differences in standing carbon stocks (Appendix 3, Additional file 1). In the N region, secondary regrowth from deforestation of at least 5 and 10 years of age resulted in mean (± s.d.) ACD of 39.4 (29.4) and 52.9 (49.5) Mg C ha^-1^, respectively. Forested areas subjected to disturbance (also known as degradation), as mapped with CLASlite, contained 70.9 (± 56.6) Mg C ha^-1^. In the S region, carbon stocks in forest regrowth varied slightly by elevation (Appendix 3, Additional file 1), but were generally between 16 and 49 Mg C ha^-1^, depending upon age and whether they were deforested or disturbed prior to assessment. In sum, aboveground carbon stocks in forest regrowth were extremely variable, but consistently much lower than in intact forest.

**Figure 5:**
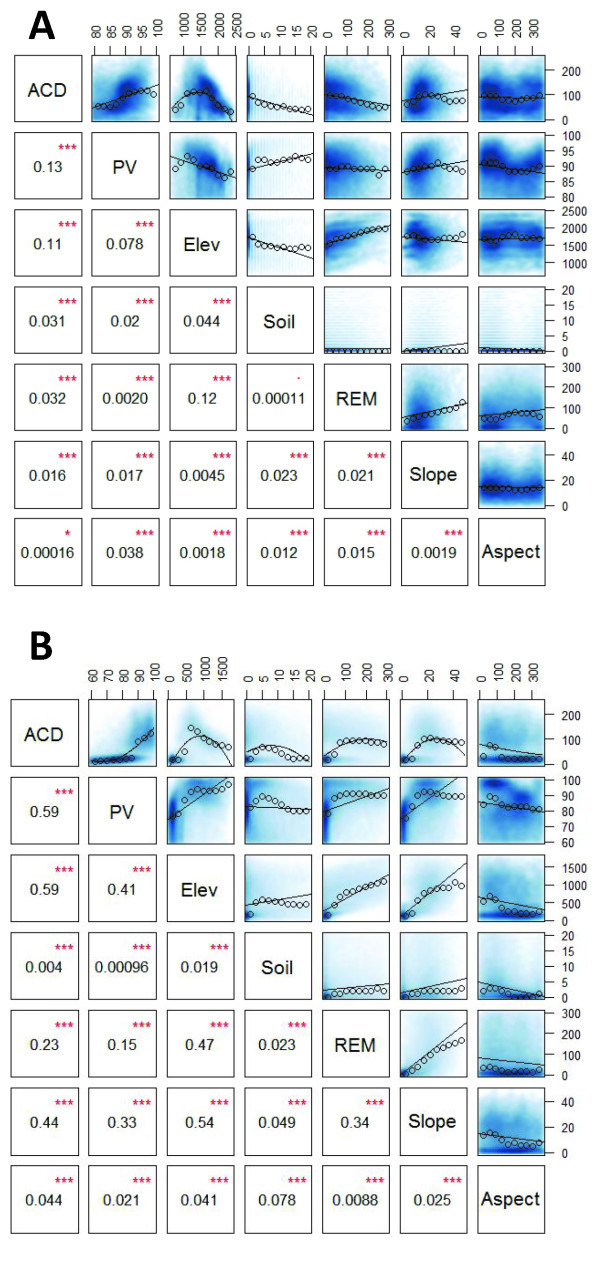
**Correlation matrices relating site factors to ACD, including elevation a.s.l. (Elev in m), fraction of photosynthetic vegetation as determined by CLASlite (PV), slope (degrees), REM (elevation above nearest drainage), fraction of soil cover as determined by CLASlite, and aspect for the (A) northern region and (B) southern region. The scatter plots display the following information: blue points indicate individual pixel values, open circles represent the median value within each of ten bins evenly spaced along the horizontal axis (these are intended to assist in visualization and do not impact model fit), lines are the best fit polynomials for the data being compared**. In the lower left portion of the matrix are the *r*^2 ^values for the best-fit polynomials; *** indicates statistical significance of *P *< 0.001.

Statistical analyses indicated that SRTM elevation and CLASlite PV cover fraction were the factors most closely associated with landscape-scale, LiDAR-based ACD variation (Figure 5). Although all variables were significantly correlated with ACD, soil fractional cover and aspect generally explained less ACD variation (< 4%). Variables explaining more than 4% of the variation, such as slope and REM in the S region, were also strongly correlated with elevation and PV cover. Based on this finding, we conducted multiple regression analyses to determine the total ACD variation that could be explained by a combination of the variables. Models including elevation and PV explained 27 and 67% of the overall variation (adjusted r^2^) in the N and S regions, respectively (Appendix 4, Additional file 1). In both regions, other factors including slope, REM, aspect and bare soil fraction did not contribute to explanatory power of the multiple regression beyond that explained by elevation and PV. As a result, these additional factors were excluded from the final regional stratification. Although the regression analyses suggested that directly modeling ACD using input terrain variables may be tractable, second-order polynomials did not provide sufficient fidelity to account for the non-linear influence of the variables (e.g., elevation and PV in the S region; Figure 5b), suggesting that a highly complicated model would be required. A stratified approach, therefore, maintained greater parsimony and potentially higher accuracy. In addition, any potential class-specific influences over ACD can be captured using a stratification approach, whereas a continuous model often smoothes them artificially.

### Regional Sources of Carbon Stock Variation

**Figure 6:**
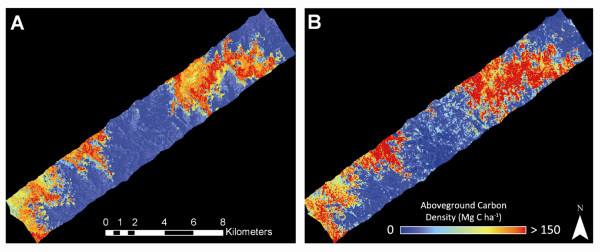
**Comparison of (A) median ACD applied to one southern humid forest landscape according to the final regional, satellite-based stratification, and (B) direct mapping of ACD using airborne LiDAR with the MCH-to-ACD model from Figure 2**. The colors, image stretch, and units are the same in both images.

Following stratification and application of the LiDAR-based ACD estimates to the full regional study extent, a comparison of LiDAR-derived ACD and the regional-scale stratified mapping of median ACD revealed the effectiveness of the stratification approach (Figure 6). Stratification by CLASlite forest cover, forest change, PV cover fraction, and SRTM elevation yielded high-resolution ACD variation that closely matched LiDAR-derived ACD. Repeated comparisons, such as that in Figure 6, suggested that scaling LiDAR data regionally in this manner is tractable if the satellite data capture the detailed variation in topographic, environmental and land-use effects at appropriate spatial scales. Our experience suggests that 1 ha or higher resolution is required to support this scaling step.

The relative error between high-resolution LiDAR-scale ACD maps and regional ACD maps was calculated as the percent difference between the square root of the median squared residual value (predicted ACD - observed ACD) and mean observed ACD. We used the median rather than mean squared residual value to compensate for highly right-skewed distributions of pixel level error. Thus, our pixel-level uncertainties reflect median uncertainties. We estimated a pixel-level uncertainty of 35% and 10% in the N and S regions, respectively, at 1 ha resolution. Greater uncertainty in the N region is caused by generally weaker relationships between environmental variables and measured ACD (Figure 5). Nonetheless, a 35% uncertainty at the pixel level is on par with errors inherent to field inventory plots [30,31], and when averaged over much larger jurisdictional scales of thousands to millions of hectares, the overall uncertainty drops to extremely low values [3,17].

Additional comparisons between regionally-extrapolated ACD and high-resolution forest cover were made by overlaying regional ACD maps on Google Earth imagery (Figure S2, Additional file 1). In one southern landscape, the largest tree crowns were clearly aligned with areas of high ACD (reds), whereas areas containing small-statured vegetation (naturally or from land use) aligned with low-to-moderate ACD (yellow-green), and no ACD (blues) estimates. In one portion of the southern montane landscape, the abrupt orographically-mediated transition from high biomass windward forests (red) to lower biomass leeward forests (orange) was evident (Figure S2, Additional file 1). This break was so distinct, it was expressed over just a few hundred meters of distance. Similar extreme gradients can be found on other tall oceanic islands where orographic effects are common [32]. Close inspection against Google imagery also revealed that suppressed ACD levels along slopes (greens-blues; Figure S2, Additional file 1) were caused either by natural landslides or human activities.

**Figure 7:**
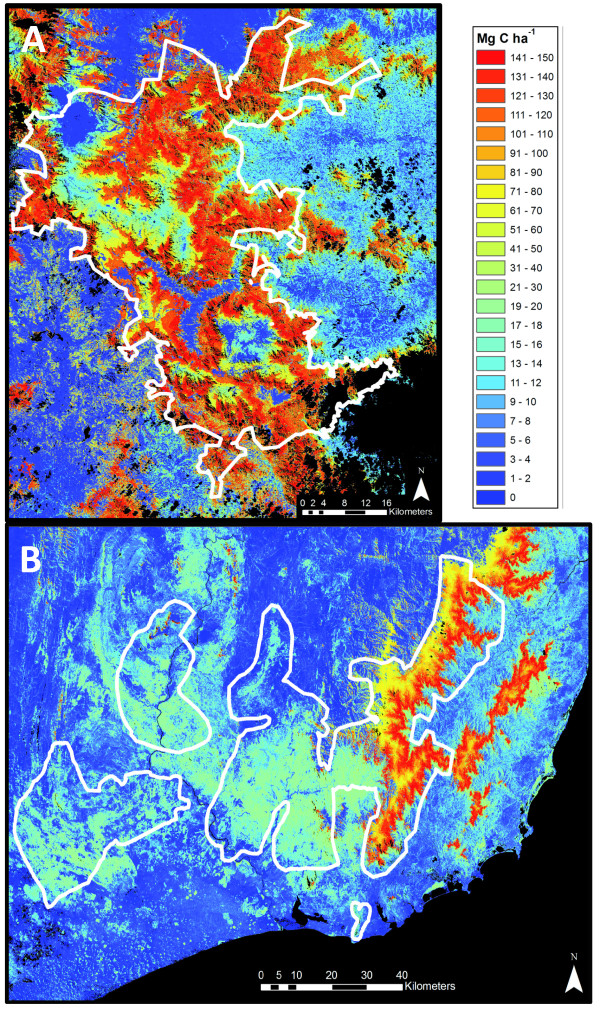
**Aboveground carbon density (ACD) across the (A) northern and (B) southern region of Madagascar**. Median ACD reflects the median value for a habitat class as measured within the LiDAR coverage for that class. Black areas are unobserved or water bodies.

The combined satellite and LiDAR-based results provided a synoptic view of aboveground carbon stocks throughout the much larger N and S mapping regions (Figure 7). In the N region, the highest ACD levels were found in upland zones farther from human activities, particularly in sub-montane hillslope positions where substrate fertility and climate harbor tall forests [19,33,34]. Sub-montane to montane forests in the S region harbored as much carbon in aboveground tissues as found in N forests, but the orographic effect confined the highest biomass areas to east-facing slopes (Figure 7b). Across both study regions, very little lowland intact forest remains, and in the south, much of the spiny forests either harbor naturally low carbon levels or have been lost to deforestation. Together these two regions are geographically important examples of the carbon landscape persisting throughout Madagascar today.

### Error Analyses

**Figure 8:**
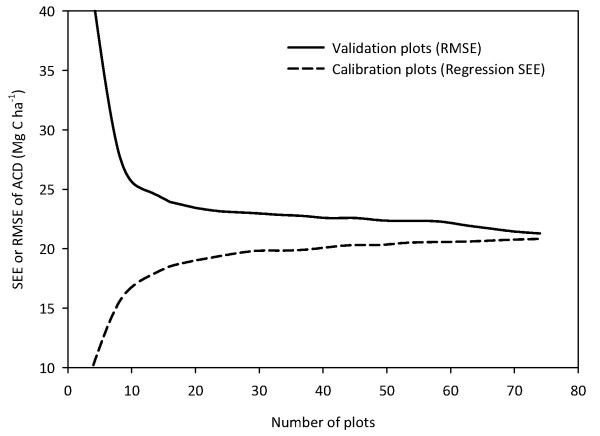
**Change in regression Standard Error of the Estimate (SEE) for a series of airborne LiDAR calibration models using multiples of 4 field plots (mean of 1000 runs per model)**. For each run, observed carbon values for a random set of 4 field plots not used in the calibration were compared to the predicted values in terms of their root mean square error (RMSE).

Simulations of both reduced numbers of field plots as well as reduced LiDAR coverage demonstrated that our sampling effort for both field plots and airborne data was effective at capturing the true variation in ACD. When considering the number of field plots needed to produce a consistent LiDAR-to-ACD calibration model, we found that regression standard error (SEE) increased rapidly up to ~ 20 plots, but stabilized within ~ 2 Mg C ha^-1 ^of the full regression SEE (21.1 Mg C ha^-1^) at just 24 of the 83 plots (Figure 8). Additional plots therefore had little effect on the regression SEE. Likewise, the ability of the simulated calibrations to accurately predict a set of 4 randomized field plots (not included in the calibration) was initially poor, but stabilized within ~ 2 Mg C ha^-1 ^of the full regression RMSE with just 24 of the 83 plots. The stabilization of the LiDAR-to-plot calibration at ~ 24 field plots is comparable to that found in a previous Amazonian study [17].

As LiDAR coverage was statistically reduced, we found that median ACD remained stable for most vegetation classes - within 10% of the true median value - until the coverage declined below ~ 300 ha (Figure S3, Additional file 1). However, for some classes, at least 2000 ha of LiDAR sampling was required to be within 10% of the true median. Conservatively, median ACD values reported for classes with > 500 ha of LiDAR coverage can be considered to have very high confidence. These large classes account for 90% and 88% of the core study areas in the N and S regions, respectively (Appendix 3, Additional file 1). These results point to the importance of obtaining thousands of samples in order to derive the true median and distribution of ACD throughout the landscape.

## Conclusions

Terrestrial carbon landscapes depict the integrated effects of many ecological and socio-economic processes occurring at spatial scales ranging from localized human activities (e.g., wood gathering) to synoptic climate-driven processes affecting vegetation growth and carbon storage. As a result, carbon maps are most informative when created over large environmental gradients while maintaining high spatial resolution. With a combination of airborne LiDAR, field calibration plots and satellite data, we mapped aboveground carbon stocks throughout two remote regions of Madagascar covering a total of 2,372,680 ha. Despite the widespread evidence of human activities throughout the regions, we found that large-scale natural controls over aboveground carbon density were driven primarily by physiography and vegetation cover. These results strongly suggest that environmental surrogates can be used to extend direct observations made by airborne LiDAR to much larger geographic scales. Our results support other work on biomass surrogates at coarser resolutions [21,35], but the high-resolution regional stratification and airborne LiDAR approach presented here yields carbon storage detail that can be validated point by point through a large region. The global and regional approaches are complimentary and could be combined in future work to improve carbon mapping at national and global levels.

Here our regionally mapped carbon stocks are the result of using very high resolution airborne LiDAR to understand the physiographic and vegetation cover controls, and then applying that knowledge outside of the LiDAR coverage using high resolution satellite data. Our previous efforts along these lines have relied on habitat classification maps, often provided by government agencies, but in this case, comparable effectiveness was achieved using freely available SRTM and Landsat imagery. The results thus suggest that verifiable, high-resolution carbon assessments can be accomplished in remote regions of the tropics. The high resolution and accuracy of the results may support new efforts to sustain and expand Malagasy forests using current voluntary and planned compliance carbon emission offset strategies.

## Methods

### Regional Pre-flight Stratification

**Figure 9:**
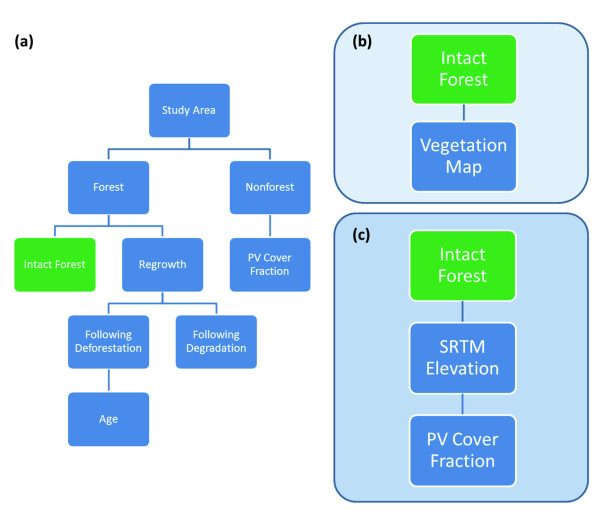
**Stepwise pathways used to stratify each study region: (a) Forest cover and regrowth mapping with multi-temporal Landsat imagery in CLASlite; (b) Combining CLASlite outputs from (a) and a classified vegetation map for regional pre-flight stratification to direct airborne LiDAR sampling; (c) Combining CLASlite outputs from (a) and elevation from the NASA Shuttle Radar Topography Mission (SRTM) in final regional stratification to capture regional ACD variation**.

Pre-flight stratification of the N and S regions was required to direct airborne LiDAR sampling (Figure 9). Following Asner [15], we developed a map of vegetation types and forest cover. The vegetation map was derived using supervised classification with a k-nearest neighbor algorithm of 12 multi-spectral images at 10 m resolution, collected between January and May 2009 by satellite SPOT 5 (SPOT Image, Toulouse, France). The derived vegetation maps yielded 8 and 10 classes in the N and S regions, respectively. The regions were simultaneously mapped for forest cover, deforestation, degradation and regrowth using the CLASlite system [36], as described in detail by Asner et al. [17]. Combining vegetation and CLASlite maps resulted in 15 and 17 classes for the N and S region, respectively. SRTM data were not used during this pre-flight stratification process, thereby facilitating an independent analysis of terrain controls over ACD later in the study, and prior to final stratification.

### LiDAR Mapping

The pre-flight stratification maps were used to direct airborne LiDAR sampling. Specifically, LiDAR polygons were placed over each study region to maximize coverage of each pre-flight stratum while attending to aircraft logistical constraints imposed by mountainous terrain and winds. A total of 12 and 10 polygons ranging in size from 2,000 ha to 12,000 ha were selected for the N and S regions, respectively (Figure 1). We used the Carnegie Airborne Observatory (CAO) to collect the LiDAR data [37]. The flights were conducted at 2000 m above ground level, providing LiDAR sampling at 1.12 m LiDAR spot spacing with a 28-degree field of view, 50 kHz pulse repetition frequency and 50% overlap between adjacent flight lines within each mapping polygon. The aircraft maintained a ground speed of 95 knots or less. LiDAR spatial errors are less than 0.15 m vertically and 0.36 m horizontally (RMSE). The total LiDAR coverage was 56,921 ha (19.6% of the core study area) and 70,780 ha (14.3%) in the N and S regions, respectively.

### Field Plot Measurements

We estimated ground-based ACD in 83 circular plots of up to 30 m radius, or 0.28 ha (Figure 1). Of these, 19 were located in N humid forests, 28 in S humid forests, and 36 in S spiny forests. We used the plots to calibrate the LiDAR data to ACD, and thus we placed the plots in a wide range of conditions, from dry to humid forests and from nearly deforested land to degraded forests to intact, closed canopy forest. This approach reduced the number of plots required overall, while providing the biophysical variation needed to calibrate the LiDAR. We used a nested sampling design in each plot, which differed depending on forest structural complexity and thus climactic zone. For humid forest plots, we included all trees > 50 cm in diameter at breast height (dbh, 1.3 m from the ground) or above buttress inside a radius of 30 m from the plot center, all trees > 20 cm dbh inside a radius of 14 m, and all trees > 5 cm dbh inside a radius of 4 m. In spiny forests, we included all trees > 5 cm dbh inside a radius of 20 m and all trees > 0 cm dbh inside a radius of 5 m. We located each plot center using a global positioning system (GPS) with a survey-grade receiver (GS50+, Leica Geosystems, St. Fallen, Switzerland). The GPS data were later differentially corrected, in most cases yielding < 1 m positional error. For some plots in the S humid forests, Leica GPS reception was poor and thus we utilized a Garmin CS+ receiver (Garmin Ltd., Olathe, Kansas) with the point averaging function enabled for several hours. When comparing Garmin and Leica data, we found that the loss of accuracy with Garmin data was 1-4 m. Allometric methods for estimating plot-level carbon stocks made use of both general [38] and recent, locally-developed models [39], as detailed in Appendix 1 (Additional file 1).

### LiDAR-to-Plot Calibration

We derived surface (top-of-canopy) and ground digital elevation models (DEM) from the airborne LiDAR data. We calculated the vertical distribution of LiDAR points above ground by binning them into volumetric pixels (voxels) of 5 × 5 m spatial resolution with 1 m vertical resolution. We then divided the number of points in each voxel by the total number of lidar points in that column, yielding the percentage of lidar points that occurred in each voxel. This organization of LiDAR data points allows the generation of several indices that have proven to be highly correlated with plot-based biomass estimates [12,29,40-43]. Previous work repeatedly demonstrated that mean canopy profile height or MCH, which is the weighted height of the lidar point cloud in a 5 × 5 kernel, is highly correlated with ACD in tropical forests [17,28,44]. Thus we regressed ground-based ACD against LiDAR MCH in the form of a power model:

(1) $$y=ax^{b}+x^{k}\varepsilon$$

where x is MCH, y is ACD, and a, b, and k are model parameters. A non-arithmetic error term (x^k^ε) was used to account for heteroscedasticity that typifies MCH-to-ACD relationships [e.g., [28]]. This approach is analogous to fitting a linear model to log-transformed variables, but avoids the need for back-transformation [45]. The model was fit using maximum likelihood in the R programming language (v.9.2, R Development Core Team 2009). We conducted additional analyses to determine the dependency of our LiDAR-to-ACD model on the number of field plots (below under *Error Analyses*). We also considered the dependency of our LiDAR-to-ACD model on the spatial resolution at which the equation was constructed (Appendix 2, Additional file 1).

### LiDAR Carbon and Terrain Analysis

We tested the degree to which terrain and forest cover variables were related to LiDAR-derived ACD. Our goal was to develop an improved method to scale up the LiDAR-based data to the regional level using freely available satellite imagery. To that end, we examined relationships between ACD and elevation data, the latter provided by NASA's Shuttle Radar Topography Mission (SRTM; 90 m resolution), as well as CLASlite forest cover data derived from Landsat imagery (30 m resolution). To improve signal via averaging, and to reduce errors due to misalignment among diverse image data types, we convolved all variables to 1 ha spatial resolution prior to analysis using a spatial averaging filter. Within areas under airborne LiDAR coverage, we considered several SRTM-derived variables, including elevation, slope, aspect, and height above nearest stream (i.e., the vertical distance from a point on the landscape to an interpolated water table, also known as a relative elevation model or REM). From CLASlite, we considered the fractional cover of photosynthetic vegetation (PV) and the fractional cover of bare soil (described in detail by Asner et al. 2009). We considered the influence on ACD of all variables-both individually and in combination-at 1 ha resolution using correlation and ordinary linear regression analyses with the R programming language (v.9.2, R Development Core Team 2009).

### Final Regional Stratification and Carbon Mapping

Final stratification was intended to utilize information obtained from the LiDAR-based environmental controls analysis, as well as the deforestation and forest disturbance results from CLASlite (Figure 9). We first segmented the region into forest, non-forest, and regrowth, derived from CLASlite analysis of Landsat images from four periods; 1990, 2000, 2005, and 2010. The CLASlite analysis provided a detailed representation of 2010 forest condition, enhanced by the consideration of past land-cover change. The broader classes of forest, non-forest and regrowth were further stratified to capture regional ACD variation. This process was guided by existing scientific knowledge of the ecology and terrain of Madagascar, as well as statistical considerations to limit over-stratification.

We stratified the forest class using the SRTM- and CLASlite-derived inputs that best predicted ACD in forested areas at the LiDAR scale. As presented in the results (*Landscape Controls over LiDAR-derived ACD*), the best predictors were SRTM elevation and CLASlite PV cover fraction. The nature of the influence of these variables on LiDAR-derived ACD suggested that a stratified approach to carbon mapping was preferable to modeling ACD in continuous fashion (see results). Thus, in the N region, we parsed the landscape into 150 m vertical increments, creating 13 elevation strata. In the S region, we created a total of 11 elevation strata with 100 m elevation increments below 500 m (characterized by dry, spiny forests and flatter terrain), and 200 m increments above 500 m in elevation (steeper, variable terrain containing the region's remaining humid forests). The two regions were further stratified by PV cover fraction with thresholds of 80-90% and 91-100% PV in the N region and 60-70%, 71-80%, 81-90%, and 91-100% in the S Region. Our approach to the S region employed a lower PV threshold to better capture dry spiny forests, which often contain lower PV fractions due to naturally open canopies and space between trees and other woody plants. The intersection of elevation and PV cover fraction strata produced 26 forested classes in the N region and 44 classes in the S region.

In both regions, we used CLASlite [36] to stratify deforested and disturbed lands (e.g., areas entering a non-forest category following previous mapping as forest cover), as well as those in regrowth following deforestation or disturbance. Here, we define disturbance as the diffuse thinning of forest cover. We split forest regrowth following deforestation into categories of greater than 5 or 10 years since the most recent detection of deforestation. We created another class for regrowth following forest disturbance detected in any year. Areas not in forest and not undergoing regrowth following deforestation or forest disturbance made up the broad non-forest class, which we stratified into five 20% intervals of PV cover fraction. In the S region, all regrowth and non-forest classes were further parsed by a 500 m elevation threshold that generally corresponds to the separation of lowland, dry spiny forests and mid-elevation humid forests. Combined, this yielded 34 total classes in the N region and 52 in the S region. Using this final stratification, we mapped ACD at 1.0 ha resolution by assigning the LiDAR-derived median carbon value for each class to the area occupied by that class across the regional maps.

### Error Analyses

We determined the sensitivity of our LiDAR ACD model to the number of field calibration plots used by fitting simulated calibration models with randomized multiples of 4 plots (~ 5% of the data), from 4 to 76 plots (up to 90% of 83 total plots) [17]. We repeated this simulated calibration 1000 times to yield a mean regression standard error of the estimate (SEE) at every interval of 4 field plots. For each run, we then assessed the ability of the model to predict (as measured by the RMSE) the carbon density of an additional set of 4 randomized field plots not included as part of the simulated calibration.

To determine the amount of LiDAR coverage needed to accurately characterize inter-class variation in ACD, we simulated reduced LiDAR coverage by successively excluding portions of all LiDAR flight boxes. To do so, we reduced the widths of all boxes beginning at a minimum width of 30 m (a single pixel of our LiDAR-derived carbon map), doubling repeatedly up to 3840 m width (an approximation of the largest width that was divisible by the pixel size of 30 m). With each successively more limited regional LiDAR sample, we recomputed median carbon for each vegetation class in both the N and S study areas, and compared the resulting estimates to those determined using the full LiDAR coverage.

## Competing interests

The authors declare that they have no competing interests.

## Authors' contributions

GA conceived of the study, analyzed data and wrote the manuscript. JK, JM, and KC analyzed data and wrote the manuscript. TK-B, RV, MR and GV collected and analyzed data. AB, LM, MC, and DK analyzed data. All authors read and approved the final manuscript.

## Supplementary Material

Additional file 1 **Appendices**. The appendix contains text, figures and tables that provide detail on satellite, aircraft, and field data collection, processing and analysis. Click here for file
