# Supplementary material for: Human and environmental controls over aboveground carbon storage in Madagascar
Source: Carbon Balance Manag. 2012 Jan 30;7:2. doi: 10.1186/1750-0680-7-2 (PMC3278681; doi:10.1186/1750-0680-7-2)
Supplement: Additional file 1 — Appendices. The appendix contains text, figures and tables that provide detail on satellite, aircraft, and field data collection, processing and analysis. [file 1750-0680-7-2-S1.DOCX]

**APPENDICES with Tables S1-S4 and Figures S1-S3**

**Appendix 1: Allometry**

We estimated aboveground carbon density (ACD) in field plots using the general allometric approach outlined by Chave et al. [38], but with improvements to reflect regional allometric variation in Madagascar [39] (Table S1). In southern forests, we used regional models to estimate ACD as a function of stem diameter, height, and wood density. For the largest trees in each plot and a sampling of trees spanning a range of diameters, height was measured in the field. For all other trees, height was estimated as a function of stem diameter following Vieilledent et al. [39]. For northern forests, field plots were located above an altitude characterized by Vieilledent et al. [39], including regions of higher precipitation (e.g., wet or cloud forests). Here we estimated ACD using a global wet forest model of Chave et al. [38]. Again, we corrected for localized height variation by directly measuring the heights of the largest trees in all plots and additional trees spanning a range of stem diameter. For the remaining trees, we produced a model relating height and diameter using Maximum Likelihood Analysis in R (R Development Core Team, 2009); the fitting method follows that described in the methods section of the LiDAR MCH-to-ACD model. For all trees across the study region, wood density values were assigned based on plant genera-level field identification using a combination of regional field-based estimates Vieilledent et al. [39] and values from a global wood density database (Table S2). For those species without a field or literature estimate, a default value of the regional mean wood density was used.

**Appendix 2: Spatial resolution of LiDAR calibration**

Airborne LiDAR mapping errors are a function of the regression error (RMSE) for a given calibration model, which itself is dependent on the spatial resolution (or plot size) used to construct the calibration [38]. In this case, the largest plots were 30m in radius (0.2827 ha), and thus our calibration error (21.1 Mg C ha^-1^) conservatively applies to a LiDAR-derived carbon maps with a spatial resolution of 0.2827 ha. With an iterative analysis across several spatial resolutions, Mascaro et al. [14] showed that, for spatial resolutions up to 1 ha, prediction errors decline in accordance with the general influence of sample size on the standard error of the mean. That is, errors decline as a function of the inverse square root of the plot size or spatial resolution. Following this, our predicted LiDAR-based error is 11.2 Mg C ha^-1^ at 1 ha spatial resolution.

**Appendix 3: LiDAR-scale statistics within vegetation classes**

Complete LiDAR-scale results for the northern region are given in Table S3, and results for the southern region are given in Table S4. Forest regrowth classes in the southern spiny forest region (e.g., below 500 m) are likely overestimated due to uncertainty caused by phenology. However, these classifications still translate into improved fidelity in the final carbon maps because these regrowth classes are associated with a much lower ACD values as directly measured by airborne LiDAR.

**Appendix 4: Controls over LiDAR-derived ACD variation**

Following the results of the correlation analyses (see Methods of main text), we conducted multiple linear regression analyses using elevation derived from NASA Shuttle Radar Topography Mission (SRTM) data (DEM) and photosynthetic vegetation coverage fraction (PV) derived from CLASlite. In the northern region, this analysis yielded the following model:

$$ACD=0.3139 DEM- 0.0001 {DEM}^{2}+3.1800 PV-404.5$$

with an adjusted *r*^2^  of 0.27 (*P* < 0.0001) and a standard error of 41.8 Mg C ha^-1^. In the southern region, the analysis yielded the following model:

$$ACD=0.2121 DEM- 0.0001 {DEM}^{2}+2.0746 PV-165.3$$

with an adjusted *r*^2^  of 0.67 (*P* < 0.0001) and a standard error of 31.5 Mg C ha^-1^.

**Table S1.** Allometric equations used to estimate height (H) in meters (m) and aboveground biomass (AGB in kg) based on stem diameter at 1.3 m aboveground (dbh) or above buttress (D in cm), and wood specific gravity (p in g cm^-3^).

| **Region** | **Par.** | **Equation** | ***r*^2^** | **Reference** |
| --- | --- | --- | --- | --- |
| N Humid | AGB | 0.0776*(D^2^*H*p)^0.94^ | 0.96* | Chave et al. 2005 [38] |
| N Humid | H | 2.4661*D^0.5659^ | 0.70 | This study |
| S Humid | AGB | exp(-1.948+1.969*ln(D)+0.66*ln(H)+0.828*ln(ρ)) | 0.90* | Vieilledent et al. [39] |
| S Humid | H | exp(1.103+0.529*ln(D)) | 0.95* | Vieilledent et al. [39] |
| S Dry | AGB | exp(-1.103+1.994*ln(D)+0.317*ln(H)+1.303*ln(ρ)) | 0.95* | Vieilledent et al. [39] |
| S Dry | H | 12.12-(12.12-1.3)*exp(-0.52*D) | 0.89* | Vieilledent et al. [39] |
| *coefficient of determination for ln(y) | | | | |

**Table S2.** Wood density values used to estimate aboveground biomass in field plots. Values are based on either samples by Vieilledent et al. [39] or a global wood density database by Chave et al. [31]. For those species with neither field nor literature estimates, we sued a default value of the regional mean wood density.

| **Genus** |  | **Wood Density (g cm^-3^)** | |  | **Number of Stems** | | |  | **Reference** |
| --- | --- | --- | --- | --- | --- | --- | --- | --- | --- |
|  |  | Humid | Spiny |  | Northern Humid | Southern Humid | Spiny |  |  |
| *Asteropeia* |  | 0.79 |  |  | 1 | 0 | 0 |  | Chave |
| *Adina* |  | 0.59 |  |  | 0 | 3 | 0 |  | Chave |
| *Albizia* |  | 0.66 | 0.56 |  | 5 | 25 | 42 |  | Vieilledent |
| *Alluaudia* |  |  | 0.31 |  | 0 | 0 | 808 |  | Vieilledent |
| *Anthocleista* |  | 0.79 |  |  | 0 | 3 | 0 |  | Vieilledent |
| *Aspidostemon* |  | 0.71 |  |  | 7 | 0 | 0 |  | Chave |
| *Bathiorhamnus* |  | 0.57 |  |  | 2 | 0 | 0 |  | Chave |
| *Bembicia* |  | 0.69 |  |  | 10 | 0 | 0 |  | Chave |
| *Breonia* |  | 0.73 |  |  | 0 | 27 | 0 |  | Chave |
| *Brochoneura* |  | 0.50 |  |  | 0 | 9 | 0 |  | Vieilledent |
| *Calophyllum* |  | 0.67 |  |  | 2 | 1 | 0 |  | Vieilledent |
| *Canarium* |  | 0.47 |  |  | 12 | 17 | 0 |  | Vieilledent |
| *Canthium* |  | 0.84 |  |  | 2 | 0 | 0 |  | Chave |
| *Capurodendron* |  |  | 0.80 |  | 0 | 0 | 3 |  | Chave |
| *Cedrelopsis* |  |  | 0.74 |  | 0 | 0 | 49 |  | Vieilledent |
| *Chrysophyllum* |  | 0.53 |  |  | 0 | 65 | 0 |  | Vieilledent |
| *Commiphora* |  |  | 0.24 |  | 0 | 0 | 976 |  | Vieilledent |
| *Croton* |  | 0.55 | 0.85 |  | 14 | 1 | 9 |  | Vieilledent |
| *Cryptocarya* |  | 0.53 |  |  | 50 | 0 | 0 |  | Vieilledent |
| *Cynometra* |  | 0.77 |  |  | 0 | 3 | 0 |  | Chave |
| *Dalbergia* |  | 0.74 | 0.74 |  | 0 | 8 | 3 |  | Chave |
| *Dichrostachys* |  |  | 0.79 |  | 0 | 0 | 4 |  | Chave |
| *Dilobeia* |  | 0.79 |  |  | 0 | 2 | 0 |  | Chave |
| *Diospyros* |  | 0.79 | 0.77 |  | 5 | 19 | 6 |  | Vieilledent |
| *Dombeya* |  | 0.27 |  |  | 52 | 16 | 0 |  | Vieilledent |
| *Ehretia* |  | 0.51 |  |  | 1 | 0 | 0 |  | Chave |
| *Elaeocarpus* |  | 0.50 |  |  | 16 | 48 | 0 |  | Chave |
| *Erythroxylum* |  | 0.68 |  |  | 30 | 0 | 0 |  | Vieilledent |
| *Eugenia* |  | 0.64 |  |  | 0 | 10 | 0 |  | Vieilledent |
| *Euphorbia* |  |  | 0.39 |  | 0 | 0 | 684 |  | Vieilledent |
| *Ficus* |  | 0.49 |  |  | 14 | 4 | 0 |  | Vieilledent |
| *Garcinia* |  | 0.69 |  |  | 1 | 0 | 0 |  | Chave |
| *Givotia* |  | 0.19 |  |  | 2 | 0 | 0 |  | Chave |
| *Grewia* |  | 0.36 | 0.69 |  | 0 | 13 | 9 |  | Vieilledent |
| *Gyrocarpus* |  |  | 0.31 |  | 0 | 0 | 98 |  | Vieilledent |
| *Harungana* |  | 0.47 |  |  | 61 | 50 | 0 |  | Vieilledent |
| *Homalium* |  | 0.77 |  |  | 24 | 19 | 0 |  | Vieilledent |
| *Hymenodictyon* |  |  | 0.48 |  | 0 | 0 | 32 |  | Chave |
| *Hyperacanthus* |  | 0.69 |  |  | 0 | 6 | 0 |  | Chave |
| *Intsia* |  | 0.69 |  |  | 1 | 0 | 0 |  | Chave |
| *Khaya* |  | 0.53 |  |  | 0 | 5 | 0 |  | Chave |
| *Macarisia* |  | 0.62 |  |  | 0 | 7 | 0 |  | Chave |
| *Magnistipula* |  | 0.81 |  |  | 0 | 4 | 0 |  | Chave |
| *Mammea* |  | 0.71 |  |  | 0 | 11 | 0 |  | Chave |
| *Mascarenhasia* |  | 0.64 |  |  | 2 | 0 | 0 |  | Chave |
| *Neobeguea* |  |  | 0.84 |  | 0 | 0 | 7 |  | Chave |
| *Noronhia* |  | 0.78 |  |  | 4 | 0 | 0 |  | Vieilledent |
| *Ocotea* |  | 0.57 |  |  | 20 | 49 | 0 |  | Vieilledent |
| *Oncostemum* |  | 0.51 |  |  | 5 | 0 | 0 |  | Chave |
| *Operculicarya* |  |  | 0.22 |  | 0 | 0 | 249 |  | Vieilledent |
| *Pachypodium* |  |  | 0.10 |  | 0 | 0 | 48 |  | Vieilledent |
| *Phyllarthron* |  | 0.89 |  |  | 1 | 0 | 0 |  | Chave |
| *Pittosporum* |  | 0.51 |  |  | 1 | 0 | 0 |  | Chave |
| *Polyalthia* |  | 0.53 |  |  | 0 | 1 | 0 |  | Chave |
| *Polyscias* |  | 0.48 |  |  | 0 | 1 | 0 |  | Chave |
| *Poupartia* |  | 0.36 |  |  | 0 | 7 | 0 |  | Chave |
| *Protorhus* |  | 0.65 |  |  | 5 | 1 | 0 |  | Chave |
| *Prunus* |  | 0.66 |  |  | 2 | 0 | 0 |  | Chave |
| *Rhus* |  |  | 0.62 |  | 0 | 0 | 1 |  | Vieilledent |
| *Scolopia* |  | 0.75 |  |  | 0 | 18 | 0 |  | Chave |
| *Securinega* |  |  | 0.80 |  | 0 | 0 | 34 |  | Vieilledent |
| *Sideroxylon* |  | 0.80 |  |  | 0 | 1 | 0 |  | Chave |
| *Stereospermum* |  |  | 0.77 |  | 0 | 0 | 16 |  | Chave |
| *Streblus* |  | 0.63 |  |  | 1 | 12 | 0 |  | Vieilledent |
| *Strychnos* |  |  | 0.73 |  | 0 | 0 | 8 |  | Vieilledent |
| *Symphonia* |  | 0.64 |  |  | 0 | 12 | 0 |  | Vieilledent |
| *Syzygium* |  | 0.68 |  |  | 60 | 1 | 0 |  | Vieilledent |
| *Tamarindus* |  |  | 0.87 |  | 0 | 0 | 2 |  | Chave |
| *Tambourissa* |  | 0.51 |  |  | 34 | 43 | 0 |  | Vieilledent |
| *Terminalia* |  | 0.62 | 0.71 |  | 1 | 0 | 21 |  | Vieilledent |
| *Tetrapterocarpon* |  |  | 0.73 |  | 0 | 0 | 4 |  | Vieilledent |
| *Trema* |  | 0.37 |  |  | 9 | 1 | 0 |  | Chave |
| *Uapaca* |  | 0.60 |  |  | 0 | 7 | 0 |  | Vieilledent |
| *Vepris* |  | 0.56 |  |  | 0 | 13 | 0 |  | Chave |
| *Vitex* |  | 0.60 |  |  | 0 | 1 | 0 |  | Chave |
| *Weinmannia* |  | 0.62 |  |  | 24 | 14 | 0 |  | Vieilledent |
| *Xylopia* |  | 0.51 |  |  | 7 | 8 | 0 |  | Chave |
| *Zanthoxylum* |  | 0.51 | 0.51 |  | 1 | 0 | 4 |  | Chave |
|  |  |  |  |  |  |  |  |  |  |
| Others |  | 0.61 | 0.35 |  | 432 | 253 | 175 |  | (mean) |
|  |  |  |  |  |  |  |  |  |  |
| Total |  |  |  |  | 921 | 819 | 3292 |  |  |
|  |  |  |  |  |  |  |  |  |  |

**Table S3.** LiDAR statistics for the northern region. ACD is aboveground carbon density (Mg C ha^-1^).

| **PV Fraction** | **DEM (m)** | **ACD Mean** | **ACD Median** | **ACD Standard Deviation** | **LiDAR Coverage (ha)** |
| --- | --- | --- | --- | --- | --- |
|  |  |  |  |  |  |
| *Forest* |  |  |  |  |  |
| 80 - 90 | Below 751 | 28.7 | 23.4 | 21.6 | 57.0 |
| 80 - 90 | 751 - 900 | 35.2 | 23.5 | 36.0 | 190.9 |
| 80 - 90 | 901 - 1050 | 48.1 | 35.9 | 48.0 | 243.9 |
| 80 - 90 | 1051 - 1200 | 75.8 | 72.7 | 60.3 | 489.2 |
| 80 - 90 | 1201 - 1350 | 71.9 | 62.8 | 62.8 | 1011.9 |
| 80 - 90 | 1351 - 1500 | 81.9 | 73.4 | 63.3 | 2317.8 |
| 80 - 90 | 1501 - 1650 | 104.8 | 109.0 | 53.5 | 2889.7 |
| 80 - 90 | 1651 - 1800 | 95.8 | 100.5 | 48.2 | 3894.2 |
| 80 - 90 | 1801 - 1950 | 75.3 | 73.5 | 40.8 | 4127.5 |
| 80 - 90 | 1951 - 2100 | 55.7 | 50.8 | 33.4 | 2958.1 |
| 80 - 90 | 2101 - 2250 | 40.0 | 38.6 | 24.8 | 1411.9 |
| 80 - 90 | 2251 - 2400 | 33.0 | 33.0 | 18.9 | 572.7 |
| 80 - 90 | Above 2400 | 22.0 | 20.3 | 17.1 | 19.7 |
| 91 - 100 | Below 751 | 44.1 | 37.5 | 32.5 | 57.6 |
| 91 - 100 | 751 - 900 | 58.2 | 54.7 | 40.2 | 198.6 |
| 91 - 100 | 901 - 1050 | 94.0 | 89.9 | 57.4 | 416.4 |
| 91 - 100 | 1051 - 1200 | 117.2 | 111.3 | 63.8 | 1261.5 |
| 91 - 100 | 1201 - 1350 | 118.5 | 113.2 | 63.7 | 1832.5 |
| 91 - 100 | 1351 - 1500 | 119.8 | 121.3 | 60.1 | 2743.4 |
| 91 - 100 | 1501 - 1650 | 118.9 | 123.1 | 55.8 | 3237.4 |
| 91 - 100 | 1651 - 1800 | 105.5 | 110.6 | 49.1 | 3239.0 |
| 91 - 100 | 1801 - 1950 | 86.1 | 85.7 | 41.5 | 2518.7 |
| 91 - 100 | 1951 - 2100 | 70.4 | 66.2 | 34.5 | 847.7 |
| 91 - 100 | 2101 - 2250 | 55.5 | 55.6 | 25.9 | 343.8 |
| 91 - 100 | 2251 - 2400 | 35.9 | 36.0 | 19.9 | 200.7 |
| 91 - 100 | Above 2400 | 14.4 | 14.8 | 13.3 | 7.1 |
|  |  |  |  |  |  |
| *Non-Forest* |  |  |  |  |  |
| 0 - 20 | Full Range | 3.2 | 0.5 | 8.2 | 353.0 |
| 21 - 40 | Full Range | 3.9 | 0.3 | 10.8 | 1141.7 |
| 41 - 60 | Full Range | 7.9 | 1.1 | 17.7 | 2195.3 |
| 61 - 80 | Full Range | 19.4 | 4.7 | 31.7 | 6257.4 |
| 81 - 100 | Full Range | 43.3 | 31.8 | 43.1 | 328.5 |
|  |  |  |  |  |  |
| *Deforestation Regrowth - 5 Years* | |  |  |  |  |
| 80 - 100 | Full Range | 39.4 | 33.4 | 29.4 | 84.0 |
|  |  |  |  |  |  |
| *Deforestation Regrowth - 10 Years* | | | | | |
| 80 - 100 | Full Range | 52.9 | 40.2 | 49.5 | 125.3 |
|  |  |  |  |  |  |
| *Disturbance Regrowth - All* | |  |  |  |  |
| 80 - 100 | Full Range | 70.9 | 54.5 | 56.6 | 42.5 |
|  |  |  |  |  |  |

**Table S4.** LiDAR statistics for the southern region. ACD is aboveground carbon density (Mg C ha^-1^).

| **PV Fraction** | **DEM (m)** | **ACD Mean** | **ACD Median** | **ACD Standard Deviation** | **LiDAR Coverage (ha)** |
| --- | --- | --- | --- | --- | --- |
|  |  |  |  |  |  |
| *Forest* |  |  |  |  |  |
| 60 - 69 | 1 - 100 | 11.7 | 11.1 | 7.1 | 1160.2 |
| 60 - 69 | 101 - 200 | 12.9 | 12.8 | 8.2 | 2037.2 |
| 60 - 69 | 201 - 300 | 10.8 | 9.8 | 8.9 | 639.8 |
| 60 - 69 | 301 - 400 | 12.3 | 10.6 | 12.3 | 562.1 |
| 60 - 69 | 401 - 500 | 13.7 | 7.7 | 21.5 | 137.8 |
| 60 - 69 | 501 - 700 | 18.3 | 8.2 | 28.0 | 120.0 |
| 60 - 69 | 701 - 900 | 21.7 | 11.8 | 29.0 | 47.0 |
| 60 - 69 | 901 - 1100 | 15.1 | 3.0 | 28.5 | 49.1 |
| 60 - 69 | 1101 - 1300 | 29.0 | 8.2 | 38.6 | 26.3 |
| 60 - 69 | 1301 - 1500 | 29.8 | 15.9 | 30.8 | 15.8 |
| 60 - 69 | 1501 + | 34.8 | 30.2 | 28.6 | 4.0 |
| 70 - 79 | 1 - 100 | 14.2 | 14.1 | 5.9 | 2454.3 |
| 70 - 79 | 101 - 200 | 17.1 | 17.1 | 7.7 | 3949.0 |
| 70 - 79 | 201 - 300 | 14.3 | 13.1 | 12.4 | 742.4 |
| 70 - 79 | 301 - 400 | 17.4 | 14.3 | 21.0 | 593.8 |
| 70 - 79 | 401 - 500 | 24.2 | 13.5 | 35.2 | 269.7 |
| 70 - 79 | 501 - 700 | 36.5 | 18.5 | 44.7 | 202.3 |
| 70 - 79 | 701 - 900 | 43.5 | 24.1 | 45.2 | 128.4 |
| 70 - 79 | 901 - 1100 | 45.9 | 30.4 | 45.2 | 114.8 |
| 70 - 79 | 1101 - 1300 | 59.5 | 59.2 | 41.7 | 93.4 |
| 70 - 79 | 1301 - 1500 | 49.6 | 51.8 | 33.0 | 75.5 |
| 70 - 79 | 1501 + | 44.7 | 39.9 | 30.2 | 30.8 |
| 80 - 89 | 1 - 100 | 18.6 | 18.1 | 7.4 | 1304.4 |
| 80 - 89 | 101 - 200 | 19.5 | 19.3 | 7.2 | 4004.2 |
| 80 - 89 | 201 - 300 | 20.5 | 18.3 | 19.2 | 1135.5 |
| 80 - 89 | 301 - 400 | 38.9 | 17.3 | 51.7 | 596.3 |
| 80 - 89 | 401 - 500 | 74.1 | 38.7 | 70.2 | 586.1 |
| 80 - 89 | 501 - 700 | 122.3 | 131.6 | 66.9 | 1076.0 |
| 80 - 89 | 701 - 900 | 111.3 | 115.6 | 50.9 | 1055.5 |
| 80 - 89 | 901 - 1100 | 94.4 | 95.9 | 37.8 | 1080.4 |
| 80 - 89 | 1101 - 1300 | 80.9 | 81.1 | 32.0 | 842.9 |
| 80 - 89 | 1301 - 1500 | 69.9 | 70.3 | 27.4 | 529.4 |
| 80 - 89 | 1501 + | 59.6 | 59.4 | 26.4 | 121.3 |
| 90 - 100 | 1 - 100 | 32.6 | 27.0 | 24.5 | 79.7 |
| 90 - 100 | 101 - 200 | 24.1 | 21.9 | 14.6 | 398.6 |
| 90 - 100 | 201 - 300 | 31.6 | 22.1 | 31.8 | 898.8 |
| 90 - 100 | 301 - 400 | 61.3 | 27.9 | 58.0 | 794.6 |
| 90 - 100 | 401 - 500 | 104.1 | 115.5 | 69.2 | 894.3 |
| 90 - 100 | 501 - 700 | 146.5 | 152.2 | 57.0 | 2618.4 |
| 90 - 100 | 701 - 900 | 135.5 | 137.1 | 47.9 | 3175.8 |
| 90 - 100 | 901 - 1100 | 109.9 | 110.1 | 38.6 | 2692.7 |
| 90 - 100 | 1101 - 1300 | 89.8 | 89.4 | 32.7 | 1994.4 |
| 90 - 100 | 1301 - 1500 | 80.4 | 79.7 | 30.7 | 1022.2 |
| 90 - 100 | 1501 + | 75.1 | 77.0 | 29.2 | 437.2 |
|  |  |  |  |  |  |
| *Non-Forest* |  |  |  |  |  |
| 0 - 20 | 0 - 500 | 2.8 | 0.9 | 4.8 | 6264.5 |
| 21 - 40 | 0 - 500 | 5.4 | 3.2 | 6.9 | 5723.0 |
| 41 - 60 | 0 - 500 | 8.6 | 6.9 | 8.9 | 8190.5 |
| 61 - 80 | 0 - 500 | 13.5 | 6.3 | 22.3 | 470.3 |
| 81 - 100 | 0 - 500 | 22.7 | 9.7 | 33.1 | 1149.8 |
| 0 - 20 | Above 500 | 1.7 | 0.0 | 6.1 | 164.6 |
| 21 - 40 | Above 500 | 5.8 | 1.3 | 12.5 | 200.7 |
| 41 - 60 | Above 500 | 14.1 | 4.1 | 25.6 | 399.6 |
| 61 – 80 | Above 500 | 48.9 | 31.7 | 47.7 | 197.4 |
| 81 - 100 | Above 500 | 67.6 | 59.1 | 53.6 | 754.1 |
|  |  |  |  |  |  |
| *Deforestation Regrowth - 5 Years* | |  |  |  |  |
| 80 - 100 | 0 - 500 | 18.8 | 18.4 | 8.5 | 3310.6 |
| 80 - 100 | Above 500 | 33.9 | 19.2 | 42.8 | 78.9 |
|  |  |  |  |  |  |
| *Deforestation Regrowth - 10 Years* | | | |  |  |
| 80 - 100 | 0 - 500 | 16.1 | 16.1 | 8.1 | 1474.1 |
| 80 - 100 | Above 500 | 21.6 | 18.4 | 22.1 | 27.5 |
|  |  |  |  |  |  |
| *Disturbance Regrowth - All* | |  |  |  |  |
| 80 - 100 | 0 - 500 | 18.1 | 17.6 | 8.1 | 843.2 |
| 80 - 100 | Above 500 | 49.2 | 30.0 | 50.1 | 30.3 |
|  |  |  |  |  |  |

**Appendix Figures**

**Figure S1.** Variation in plot-level aboveground carbon density (ACD) according to general forest type in Madagascar.

**
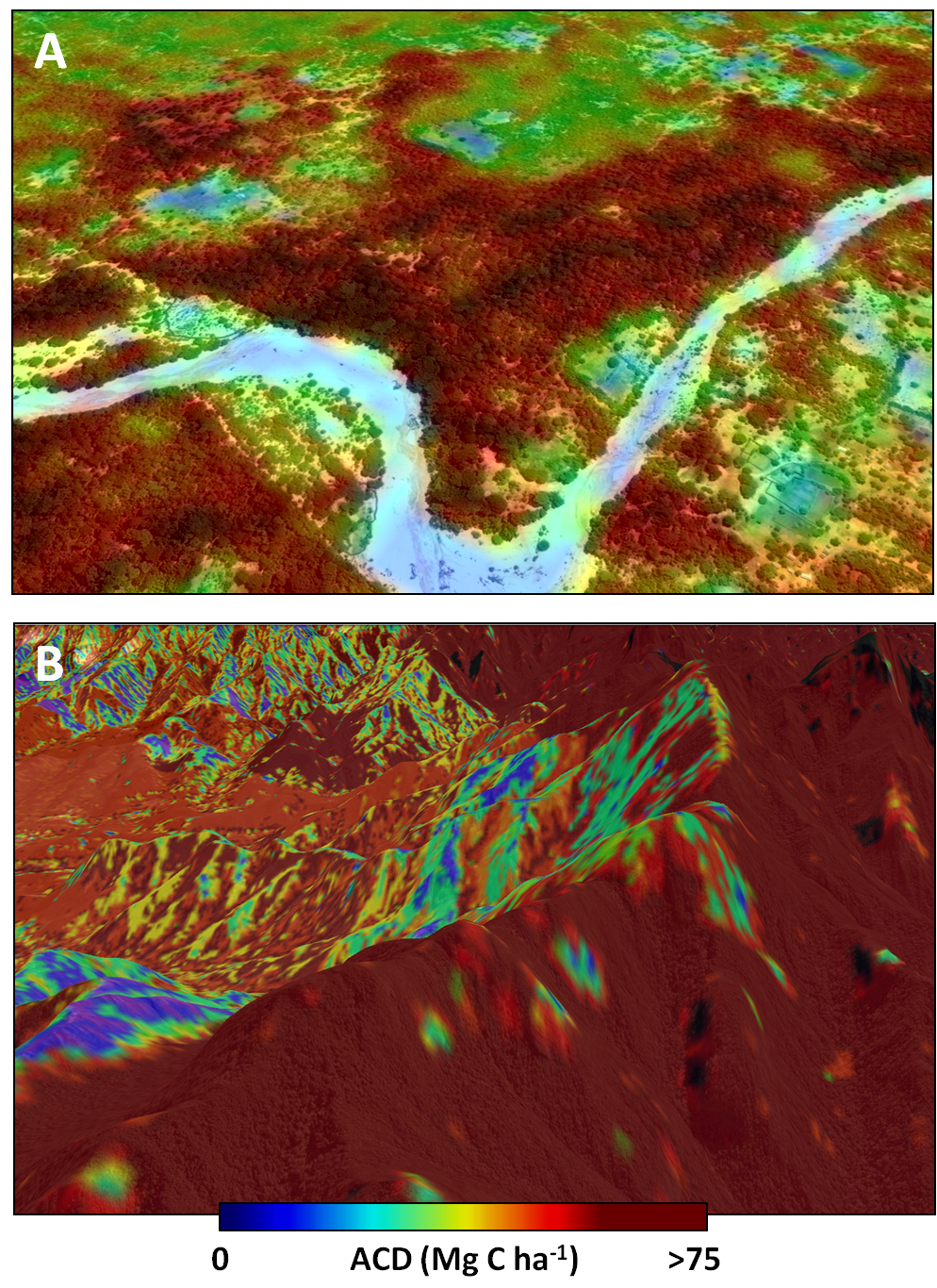
**

**Figure S2.** Comparisons of regionally extrapolated aboveground carbon density (ACD) overlaid on high-resolution vegetation imagery from Google Earth. Notice how the regional mapping estimates of high (red) to low (blue) carbon stocks align spatially with the observed distribution of trees in the Google Earth imagery.

**Figure S3.** Change in normalized median carbon density (on a percentage basis) derived from airborne LiDAR relative to the total LiDAR coverage for 26 vegetation classes in the N region and 44 in the S region. Thus the median carbon density for successive LiDAR extents was compared based on the percentage by which they differed from the median value at full coverage.
